# High-permeability vacuum membrane distillation utilizing mechanically compressed carbon nanotube membranes†

**DOI:** 10.1039/d1ra08042c

**Published:** 2021-12-20

**Authors:** Woosang Jung, Younjeong Choe, Taewoo Kim, Jong G. Ok, Hong H. Lee, Yong Hyup Kim

**Affiliations:** Department of Aerospace Engineering, Seoul National University Seoul 08826 Republic of Korea yongkim@snu.ac.kr; Department of Mechanical Engineering, Incheon National University Incheon 22012 Republic of Korea; Department of Mechanical and Automotive Engineering, Seoul National University of Science and Technology Seoul 01811 Republic of Korea jgok@seoultech.ac.kr; School of Chemical and Biological Engineering, Seoul National University Seoul 08826 Republic of Korea honghlee@snu.ac.kr

## Abstract

Membranes for membrane distillation (MD) are mostly made of polymeric and ceramic materials. We demonstrate here that the laterally-compressed, vertically-aligned CNTs (VACNT) obtainable from a CNT forest are an excellent membrane material for vacuum membrane distillation (VMD). The VACNT structure provides interstices between CNTs for extracting vaporized water molecules, while efficiently filtering the impurity salts. The VACNT membrane is shown to deliver excellent performance when tested for the desalination of 3.5 wt% NaCl water solution, as exemplified by the permeability of 68 LMH (liter per square meter per hour) achieved at the salt rejection of over 99.8% at 65 °C. We also demonstrate that the VACNT membrane performance can be maintained with time with the aid of a simple cleaning procedure, which bodes well for a long lifetime of the membrane for VMD application.

## Abstract 2

High-permeable vacuum membrane distillation by applying vertically aligned carbon nanotube for the first time.

## Graphical abstract

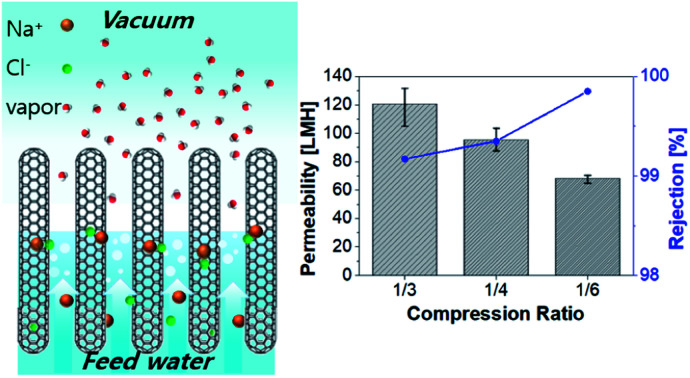

## Introduction

For decades, carbon nanotubes (CNTs) have been a workhorse for many applications in diverse fields including mechanics, electronics, photonics, and others.^1–3^ CNTs have also been utilized for water treatments. A notable feature of CNTs is that fluid can go through the tubes at a rate much faster than is possible through other porous structures with similar pore sizes of several nanometers, as confirmed by molecular dynamics simulation results.^4,5^ The permeability of CNTs significantly surpasses the level theoretically suggested by the Knudsen model.^6–8^ These phenomena may be due to the OH bond in a water molecule that combines with the inner wall of the CNT to form a depletion layer, which helps induce frictionless water movement.

The interstices between nanotubes in a vertically grown CNT forest have been utilized as membrane pores. The CNT forest grown by chemical vapor deposition (CVD) can be compressed to yield a structure that is well-aligned vertically.^9,10^ In fact, this vertically-aligned CNT (VACNT) structure has been used as a membrane.^9,11^ When the VACNT structure is used as a membrane, water passes through the interstices between CNTs with the assistance of adequate pressure. The permeability of pure water through the VACNT membrane has been demonstrated to reach 13 200 LMH per bar (liter per square meter per hour per bar) when the interstices between CNTs were controlled and the friction of water molecules reduced.^12^

This VACNT providing quite a high water permeability could be a good candidate material for the membrane distillation (MD) in which the water vapor is separated through the membrane from impurity species such as ionic salts. Among various MD techniques, vacuum membrane distillation (VMD) has the advantages of low operating temperature and rapid transport.^13,14^ However, most of the VMD materials have been thus far limited to hydrophobic polymers and ceramics, typically in the hollow fiber membrane configuration.^15–20^ The intrinsic pore-aligned structure and favorable water transport properties could make the VACNT membrane highly suitable for novel VMD systems.

**Fig. 1:**
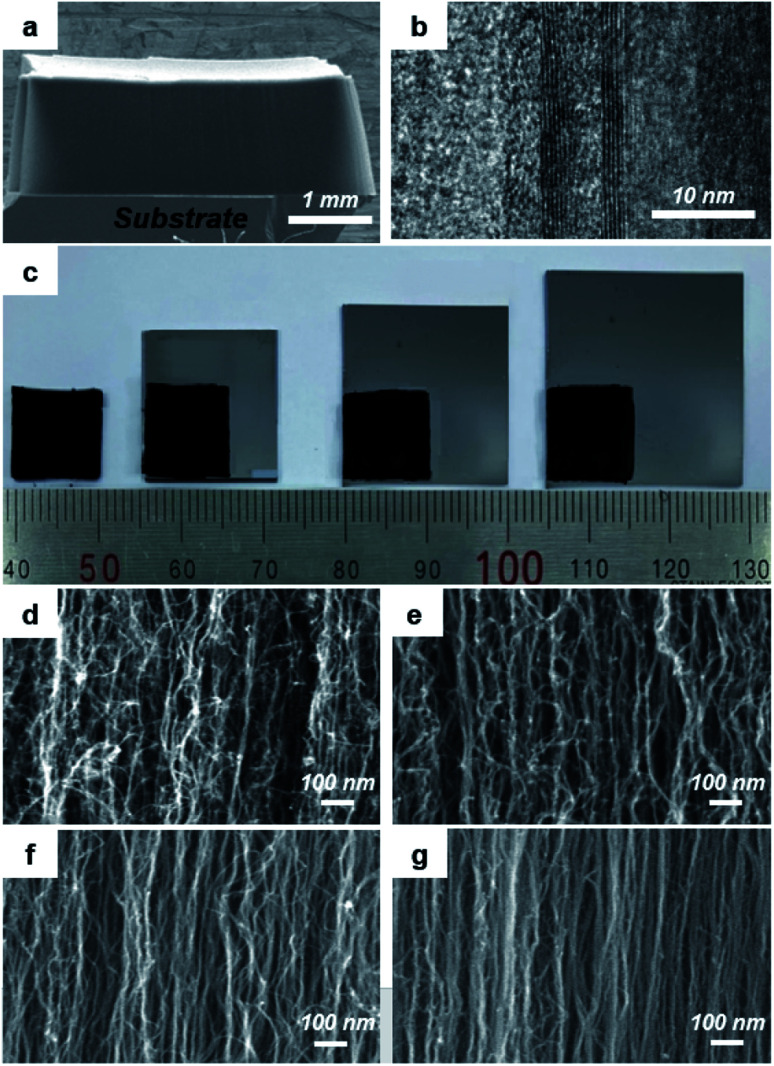
(a) Super-grown VACNT forest with the height up to 1.2 mm. (b) Representative TEM image of an individual CNT strand. (c) Optical image comparatively showing the VACNT forests with the mechanical compression ratios of 1 (no compression), 1/3, 1/4, and 1/6, from left to right, and their corresponding SEM images for the sidewall structures: (d) 1, (e) 1/3, (f) 1/4, and (g) 1/6 compression ratios.

We develop here a highly permeable and robust VACNT-based VMD system. The simple but effective mechanical compression^10^ of the as-grown CNT forest in the lateral direction leads to the VACNT membrane that can sustain its intrinsic structure against the pressure by the increased density.^21,22^ More importantly, the pore density of the VACNT membrane can be specifically controlled by adjusting the mechanical compression ratio, *i.e.*, the ratio of the compressed CNT forest area to the as-grown area (see Fig. 1c), thereby enabling tailoring of the VMD properties for various applications.

We carried out desalination of 3.5 wt% NaCl-dissolved water by VMD to demonstrate the remarkable performance of the VCANT membrane. We further demonstrate that this VMD performance can be maintained for a long time by the periodic cleaning procedure involving simple rinsing in deionized (DI) water.

## Results and discussion

The vertical CNT forest was grown on a Fe-coated Si wafer by the water-assisted CVD^12^ in which the vapor removes amorphous carbon to help extend the catalyst life, thereby enabling the ‘super-growth’ of CNTs to the millimeter scale (∼1.2 mm in average in this work; Fig. 1a). Detailed procedure is given in the Experimental section. Transmission electron microscopy (TEM) images of 40 CNT strands were taken, as shown in Fig. 1b, to determine the range of the CNT diameter and that of the wall number. The CNT diameter fell between 6 nm and 11 nm with an average of 8.8 nm and the wall number ranged from 2 and 5 with an average of 3.3. The full statistical data are provided in Fig. S1 in the ESI.† The aspect ratio of CNTs was calculated to be 1.36 × 10^5^.

The areal density, which is the number of CNTs per unit area, was calculated with the data on the thickness, wall number, and density of the CNT forest. The areal density of the CNT forest can be expressed as follows:^23^1
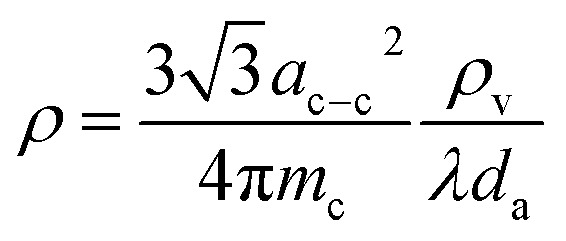
where *ρ* is the areal density of CNTs, *a*_c–c_ is the distance between carbon atoms (1.44 Å), m_c_ is the mass of carbon atom (1.993 × 10^−26^ kg), *ρ*_v_ is the density of the CNT forest, *λ* is the number of CNT walls, and *d*_a_ is the diameter of the CNTs. The density of CNT forests was measured to be 14.58 kg m^−3^, averaged from multiple samples grown in various sizes. Putting these values into eqn (1), the areal density of the CNT forest was obtained to be 2.160 × 10^10^/cm^2^. Assuming that the interstices between CNTs are of circular shape, the pore size (*i.e.*, diameter) of the as-grown CNT forest was calculated to be 76.27 nm.

The as-grown VACNT structure can be mechanically compressed^10^ for the VMD membrane. The pore size, which is one of the most important parameters dictating the VMD performance, can also be controlled through mechanical compression. After the CNT forest was separated from the Si wafer, it was compressed laterally to obtain the controlled compression ratio, which is defined as the ratio of the compressed area to the original area. The schematic illustration of the mechanical compression procedure is presented in Fig. S2 in the ESI.†Fig. 1c shows the mechanically compressed VACNT samples with the compression ratios of 1/3, 1/4, and 1/6, along with the as-grown (non-compressed) one as the reference. The CNT growth area was varied for different compression ratios to yield the same final compressed area of 1 × 1 cm^2^, as shown in the figure where the grey area was the original CNT area. Fig. 1d–g show the scanning electron microscope (SEM) images of the sidewall structures of the compressed VACNTs for various compression ratios. It can be seen that the CNT strands are compacted by compression, reducing the interstices between the strands. The pore sizes can be calculated according to the compression ratios, which were 43 nm, 37 nm, and 30 nm, respectively, for the VACNT structures compressed with the ratios of 1/3, 1/4, and 1/6. Pore distribution could be confirmed using the AFM (Atomic Force Microscopy) image of CNTs, and the average size was similar to the calculated value (Fig. S3†).

**Fig. 2:**
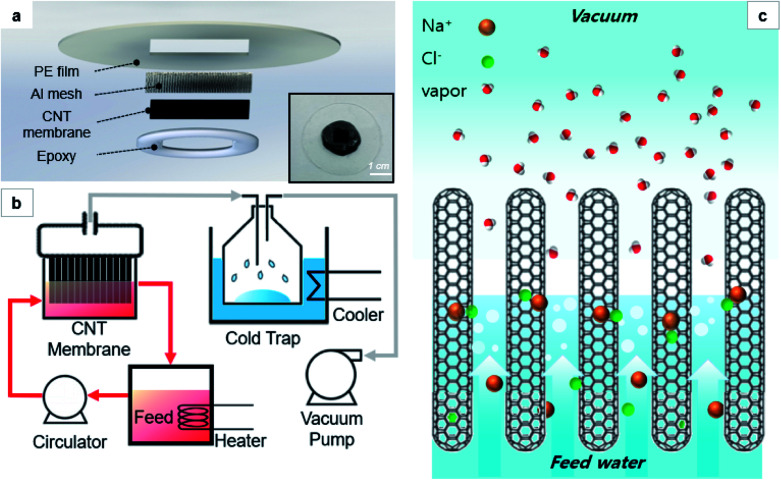
(a) Schematic drawing of the VACNT membrane assembly. The inset shows the optical photograph of an actual sample. (b) Experimental setup of the VMD using the VACNT membrane. (c) Schematic illustration of the VMD process of the NaCl solution through the VACNT membrane.

Fig. 2 shows the VMD process using the mechanically compressed VACNT membrane and its mechanism. Fig. 2a reveals the membrane assembly. In the assembly, the VACNT membrane is sandwiched between the aluminum mesh and the epoxy adhesive. Above the Al mesh is a square-holed polyethylene (PE) film. The full assembly including the stainless-steel jacket and the connector to the vacuum line is additionally shown in Fig. S4 in the ESI.†Fig. 2b depicts the overall experimental setup. Connected to the vacuum outlet through the cold trap, the membrane assembly is dipped into the 3.5 wt% NaCl solution (simply ‘feed water’ hereafter), which is heated to a controlled temperature and circulated. This heated feed water can readily penetrate through the VACNT membrane, which is facilitated by good wetting of the pores between CNTs, as discussed in detail later.

The VMD process using CNT membrane works by a new mechanism suggested in Fig. 2c. The heated feed water can readily penetrate through the VACNT membrane, which is facilitated by good wetting of the pores between CNTs, as will be examined later. When the vacuum is applied, pore wetting of the CNT membrane occurs, and feed water enters the interstices of CNTs. Inside the narrow interstices is a vacuum of about 5 kPa, where the boiling point is about 30 °C, and the feed water is thus over the boiling point. Since the vaporization rate is greater than the water transport rate at which water intrudes the pore, the water entering the CNT passes through the membrane as a vapor. Water does not pass through the membrane despite pore wetting but vaporizes and moves through in a gaseous state. Therefore, membrane distillation is possible. Experiments to elucidate this will be presented in detail later.

**Fig. 3:**
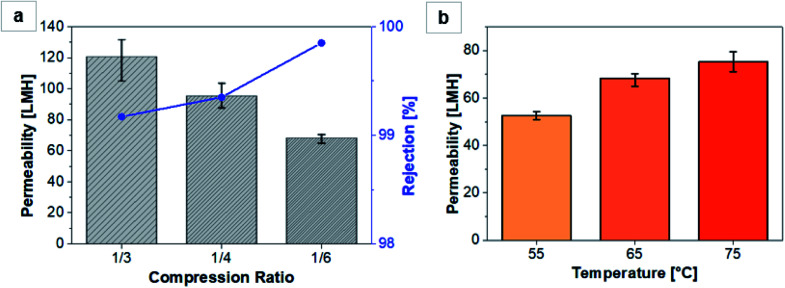
(a) Permeability and rejection characteristics of the VACNT membranes with varied compression ratios. (b) Permeability of the feed waters of varied temperatures through the 1/6-compressed VACNT membrane. Notably, the rejection was over 99.8% regardless of the feed water temperature.

The VMD performance of the VACNT membrane was evaluated with the water solution containing 3.5 wt% NaCl. It is noted here that the non-compressed, as-grown VACNT structure could not withstand the VMD operation, presumably stemming from the weak bonding of van der Waals type between the as-grown CNT strands. The mechanical compression can generally improve the mechanical strength of the VACNT structure. The rigidity could be compared by performing the compression test of CNTs according to the compression ratio.^24^ As the compression ratio increased, the compression stress also increased to 7.46, 7.82, and 11.64 MPa, with a similar strain of 0.0086, confirming the increase in mechanical strength due to compression (Fig. S5†). Fig. 3a depicts the VMD permeability and the salt rejection obtained when the salted water at 65 °C was fed to the VACNT membranes compacted to various compression ratios of 1/3, 1/4, and 1/6 (simply ‘1/3 membrane’ and so on, hereafter). The result shows that the permeability decreases with an increasing level of compression whereas the salt rejection increases, which is consistent with the typical separation behavior of better purity at the expense of lower throughput. The decrease in the permeability can be explained by the liquid entry pressure. The entry pressure, which is the minimum pressure that a liquid can pass through the membrane, is inversely proportional to the pore size.^25,26^ In addition, in Fig. S6 of the ESI,† gas permeance was tested according to the compression ratio to confirm the effect of gas transport of CNTs. All had very high values but gas permeance increased with compression ratio.

On the other hand, the rejection increases due to the smaller pore size. The 1/3 membrane had an excellent permeability of 120 LMH, and that of the 1/6 membrane was as high as 68 LMH. While the reasonable rejection of 99.2% was obtained for the 1/3 membrane, the 1/6 membrane exhibited a very high rejection of 99.85%. These results suggest that the permeability and rejection can be practically regulated depending on the targeted use, simply by controlling the compression ratio of the VACNT membrane without resorting to the case-by-case membrane fabrication of specific pore size.

It is noted here that CNTs have been used mostly as an additive to polymeric membranes to enhance the performance in MD applications^27^ by increasing hydrophobicity and porosity^28,29^ or roughness.^30^ An exception was the CNT buckypaper that was obtained by dispersing CNTs in a solvent, filtering the CNTs, and then drying to form a mat of CNTs. The buckypaper was coated with a polymeric material on both sides by sputtering. This buckypaper membrane delivered a permeation of 7.5 LMH with a rejection level of 99.9% at 95 °C.^31^

The feed water temperature can also affect the permeability. Fig. 3b shows the permeability of the 1/6 membrane for the water feeds at different temperatures. As the feed water temperature increases, the transport and evaporation of water molecules can be more active, leading to a higher permeation. At 75 °C condition, a high flux of 75 LMH could be achieved, while maintaining the rejection higher than 99.8%. A reasonable permeability of 52 LMH with >99.8% rejection can be realized at an even lower temperature of 55 °C, demonstrating the generally high permeability of the VACNT membrane.

**Fig. 4:**
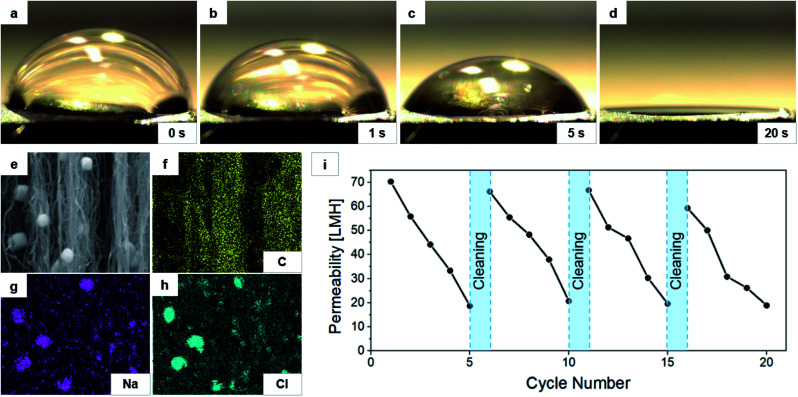
(a–d) Contact angles of the water droplet dispensed on top of the 1/6-compressed VACNT membrane surface, measured after (a) 0 s, (b) 1 s, (c) 5 s, and (d) 20 s from dispensing. (e) SEM image of the side view of the 1/6-compressed CNT membrane after VMD experiment and its corresponding EDX mapping images for the elements of (f) C, (g) Na, and (h) Cl. (i) Permeability measurement results for 20 times-repeated VMD operations of the 1/6-compressed CNT membrane. In every five operations, the DI rinsing was applied as marked in the plot.

The key to the excellent permeability may have to do with the pore wetting of CNTs, as mentioned before. When the VACNT structure contacts water, it absorbs the water, and wetting occurs.^32,33^ This behavior can be confirmed by observing the water droplet dispensed on top of the VACNT structure. Fig. 4a–d show the change of the water droplet over time on the 1/6 VACNT surface. The contact angle of the water droplet was ∼84° at the initial dispensing but shortly decreased, and the water droplet was fully absorbed within 20 seconds in our experiment. The other contact angles are a bit smaller: 55° for 1/3 and 68° for 1/4 membrane. They were on the same trend toward 0° in 30 seconds. This behavior suggests that the pore wetting by the feed water is activated in the VACNT membrane during VMD. Once wet, the CNT membrane becomes very hydrophilic, and the contact angle is close to 0°.

In addition, the water ascent to the top of the CNTs through the interstices and vaporization on the VACNT membrane were confirmed by the additional visualization experiment described in Fig. S7 in the ESI.† Inserting the glass channel in between the aluminum mesh and the PE film and taking the serial photos through the glass window during the VMD process. In Fig. S7e,† the pressure reaches 5.1 kPa, and distillation is in progress, but water is no longer visible inside the glass. Water entering the membrane by wetting evaporates inside the CNT and becomes a vapor, which is invisible. Through this, the pore wetting mechanism of the CNT membrane can be confirmed.

The mechanism also can be confirmed through membrane rejection, other than the visualization method. CNT membrane has a rejection of at least 99.2%, and the salt crystals remain inside the VACNT membrane after the experiment. Fig. 4e–h show the SEM and energy-dispersive X-ray spectroscopy (EDX) mapping images of the sidewalls of freeze-dried VACNTs after the VMD experiment. Clearly seen are the cuboid crystals observed in the SEM, which consist of Na and Cl, as revealed by EDX. Na^+^ and Cl^−^ ions that enter the membrane along with water are remain crystallized as the water evaporates.

Effective capture of salt crystals within the VACNT membrane can possibly reduce the VMD performance over time. A cyclic VMD operation was performed to evaluate the lifespan of the 1/6 membrane. One experiment was carried out for 10 minutes, and the membrane was continuously immersed in water. The membrane was taken out, washed with running water, and immersed in DI water for 5 minutes for cleaning. The amount of water that came out at one time was less than 1 mL as the process was carried out for a short time. Fig. 4i shows the permeability measured 20 times for 20 cycles, where the membrane assembly was cleaned by simple DI rinsing after every five measurements. It is apparent from the figure that the permeability decreased due to the accumulated salt in the pores and that the reduced permeability was restored by the cleaning procedure. These results bode well for prolonged use of the membrane for VMD applications.

## Experimental

### Fabrication mechanically compressed VACNT membranes

The detailed process of the VACNT super-growth can be found in the literature.^12^ Briefly, VACNTs are grown by heating a Si wafer coated with the 1 nm-thick iron at 810 °C for 16 minutes while flowing Ar, C_2_H_2_, and water vapor. The as-grown VACNT was separated from the wafer by using a razor and was mechanically compressed as schematically illustrated in Fig. S2 in the ESI;† after the as-grown CNT forest is placed on a glass plate, the two sides are fixed in an L-shaped block with a slightly lower height than that of the CNT forest. Then with the upper surface covered with another glass piece, the two surfaces are compressed sequentially using Teflon blocks to become 1 × 1 cm^2^. The initial sizes of the as-grown VACNT wafers were 1.7 × 1.7, 2 × 2, and 2.5 × 2.5 cm^2^ so that the final VACNTs compressed to the ratios of 1/3, 1/4, and 1/6, respectively, could all have the same areas of 1 × 1 cm^2^.

### VMD setup and experiment

The configuration of the entire membrane system, including the VACNT membrane, is shown in detail in Fig. S4 in the ESI.† The stainless steel parts (Fig. S4a†) were made of SUS 303 materials. The PE film (200 μm, LDPE, Sigma-Aldrich), aluminum mesh (pore diameter 100 μm, Disk Fine coffee filter, DONG SANG HARDWARE Ltd.), VACNT membrane, and epoxy glue (Twin Tube, J-B Weld Company, USA) were used to compose the VACNT membrane assembly shown in Fig. S4b and c† as well as Fig. 2a. The exposed CNT area surrounded by the epoxy (see Fig. S4b†) for the effective VMD channel was 0.6–0.8 cm^2^. The 3.5 wt% NaCl solution was used as the feed water which was circulated at the speed of 5 mL s^−1^ with the temperature of 55–75 °C maintained throughout the VMD process and the experiment was carried out for 30 minutes.

Then the stainless steel-mounted VACNT membrane assembly was in contact above the surface of the feed water. The vacuum was applied upward through the vacuum line connecter, gradually increasing from low vacuum to 5.1 kPa. The VMD-ed water vapor was collected at the cold trap maintained at −5 °C. The cleaning (discussed above with Fig. 4i) was carried out by gently rinsing the membrane with DI water and immersing the membrane in DI water for 5 minutes.

### Characterization

Scanning electron microscope (SEM) images were taken by using a Hitachi S-4800. Transmission electron microscope (TEM) images were obtained by using a JEM-2100F (JEOL Ltd.). Energy dispersive X-ray (EDX) images were taken by using the MERLIN Compact system (ZEISS). All measurements were repeated three to five times and averaged, and were plotted with the corresponding error bars.

## Conclusions

In summary, we have demonstrated that the mechanically compressed VACNT membrane enables the VMD of high permeability and rejection for impurity salt-containing water. The controlled mechanical compression of the as-grown VACNT structure can regulate the areal density and pore size of the resulting VACNT membrane, which can consequently control the permeability and rejection for desired performance. By performing the VMD through the 1/6-compressed VACNT membrane, the high permeability and rejection of 68 LMH and 99.85%, respectively, are achieved for the 65 °C feed water. This remarkable VMD performance may be attributed to the effective pore wetting and salt filtering characteristics of the CNTs. The VACNT membrane can be used for a long time by simple periodic cleaning, suggesting that many practical VMD systems can benefit from this work.

## Author contributions

Woosang Jung: methodology, experiment, characterization, analysis, resources, and writing. Younjeong Choe: investigation, and visualization. Taewoo Kim: analysis, conceptualization and methodology. Jong G. Ok: supervision, review, and editing. Hong H. Lee: proposing, supervision, and writing. Yong Hyup Kim: supervision and analysis.

## Conflicts of interest

There are no conflicts to declare.

## Supplementary Material

RA-012-D1RA08042C-s001
