# Supplementary material for: High-permeability vacuum membrane distillation utilizing mechanically compressed carbon nanotube membranes
Source: RSC Adv. 2021 Dec 20;12(1):201–6. doi: 10.1039/d1ra08042c (PMC8978618; doi:10.1039/d1ra08042c)
Supplement: RA-012-D1RA08042C-s001 [file RA-012-D1RA08042C-s001.pdf]

Electronic Supplementary Material (ESI) for RSC Advances.
This journal is © The Royal Society of Chemistry 2021

Electronic supplementary information

High-permeability vacuum membrane distillation utilizing mechanically compressed carbon nanotube membranes

Woosang Jung[a], Younjeong Choe[a], Taewoo Kim[b], Jong G. Ok[c,*], Hong H. Lee[d,*], and Yong Hyup Kim[a,*]

[a] Department of Aerospace Engineering, Seoul National University, Seoul 08826, Republic of Korea.

[b] Department of Mechanical Engineering, Incheon National University, Incheon 22012, South Korea.

[c] Department of Mechanical and Automotive Engineering, Seoul National University of Science and Technology, Seoul 01811, Republic of Korea.

[d] School of Chemical and Biological Engineering, Seoul National University, Seoul 08826, Republic of Korea.

*Corresponding authors:

Prof. Dr. Jong G. Ok: jgok@seoultech.ac.kr.

Prof. Emer. Dr. Hong H. Lee: honghlee@snu.ac.kr.

Prof. Dr. Yong Hyup Kim: yongkim@snu.ac.kr.

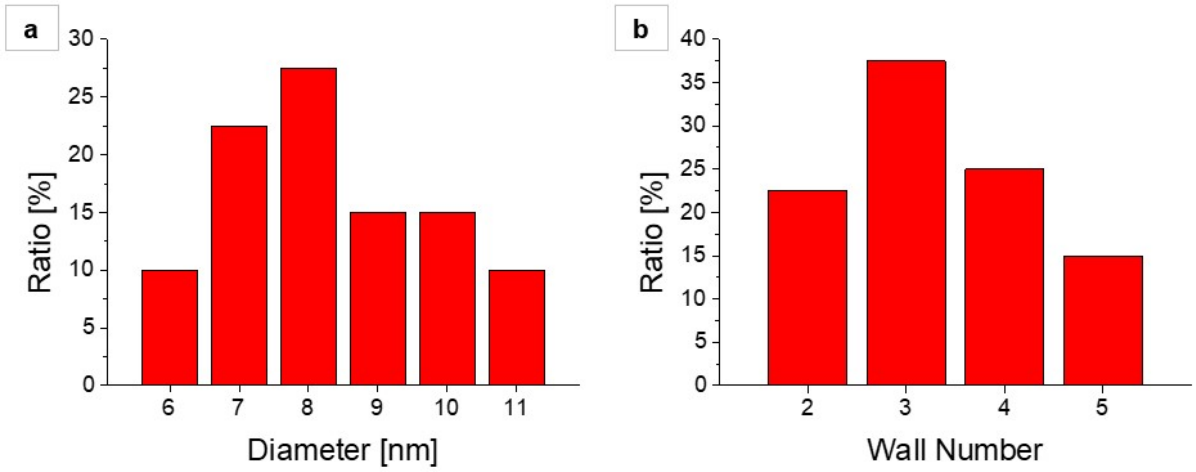

Fig. S1 Distribution of the diameters (a) and wall numbers (b) of as-grown carbon nanotubes (CNTs).

The diameters and wall numbers of CNTs were statistically investigated through the TEM imaging of 40 CNT strands. The measured values were plotted in **Fig. S1**. As can be seen in the plots, both the CNT diameters and wall numbers follow the Gaussian distribution.

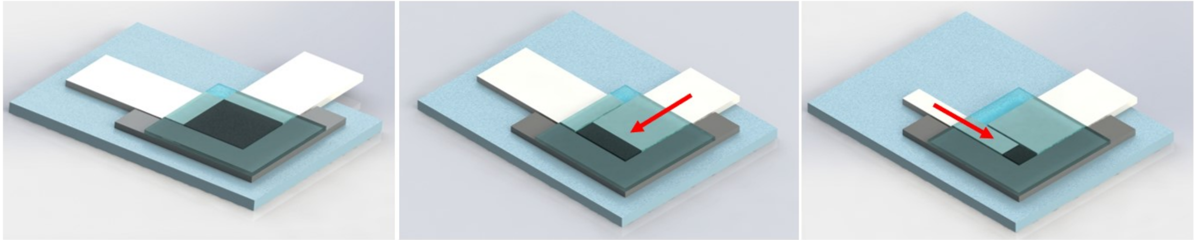

Fig. S2 Step-wise schematic drawings of the CNT compression procedure.

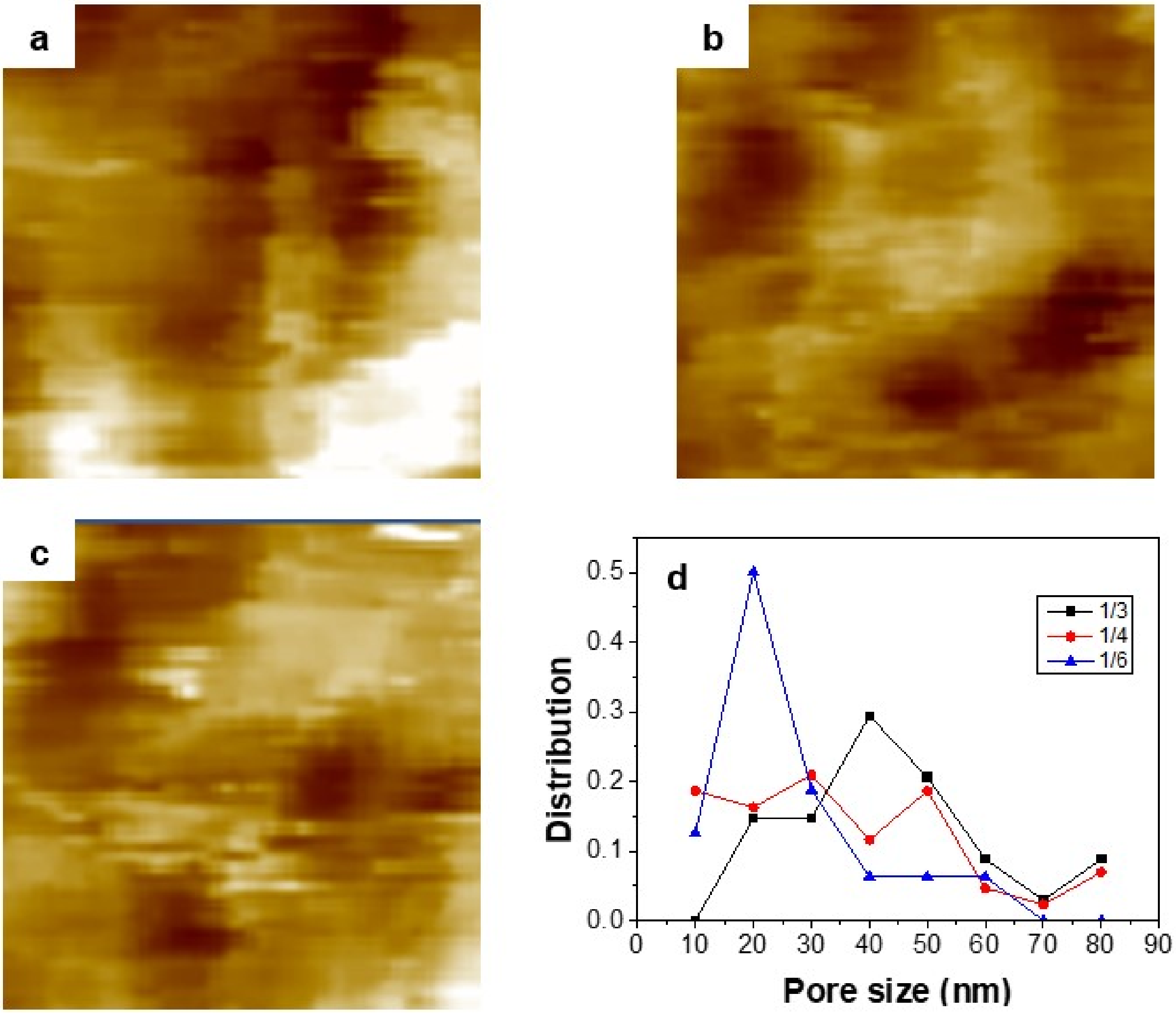

Fig. S3 AFM images according to the compression ratio (a-c) and pore distribution of membranes (d).

In AFM images, the empty part without CNT was recognized as a pore, and the size distribution was represented. The scan size of the images is 200 × 200 nm^2.

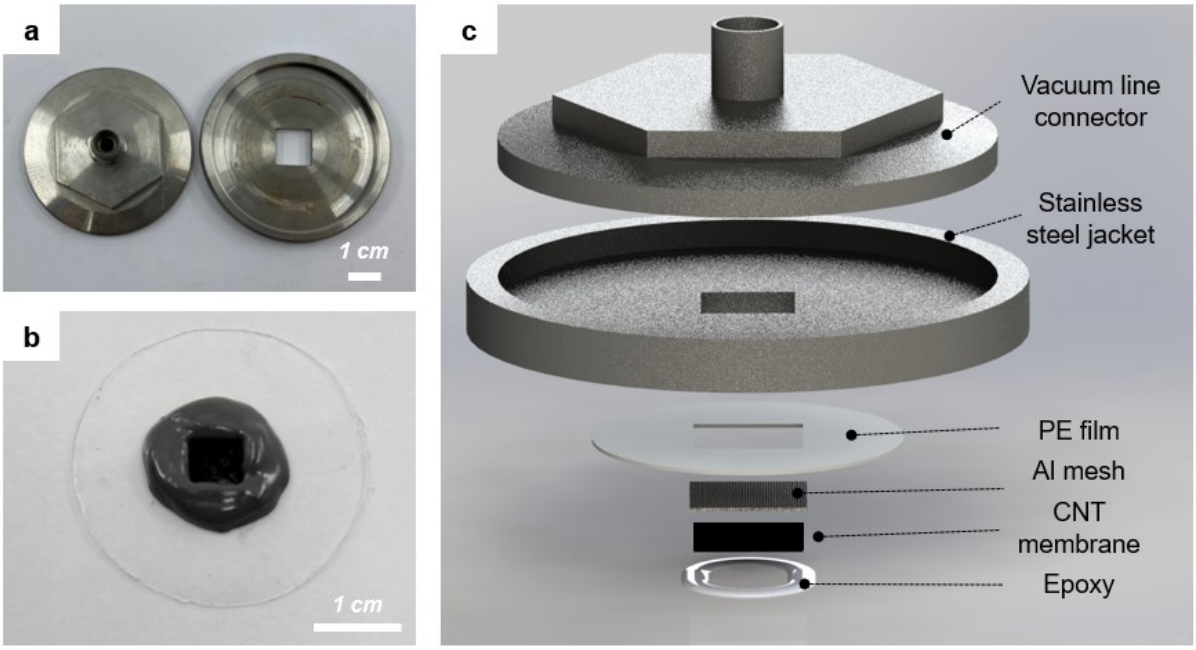

Fig. S4 Membrane system for the VMD experiment. (a) stainless steel parts including the vacuum line connector (left) and the square-holed jacket for the feed water bath (right). The vertically-aligned (VA) CNT membrane assembly consisting of the polyethylene (PE) film, VACNT structure, aluminum mesh, and epoxy glue (b) is attached to the stainless steel jacket, as illustrated in (c).

Fig. S4 shows the overall structure of the membrane assembly. The vacuum line connector and feed water jacket have threads to combine with each other. The VACNT membrane assembly (**fig. S4b**) is tightly fixed underneath the jacket using an aluminum tape. The entire system is brought into contact with water with the membrane facing down, and the vacuum is applied upwards.

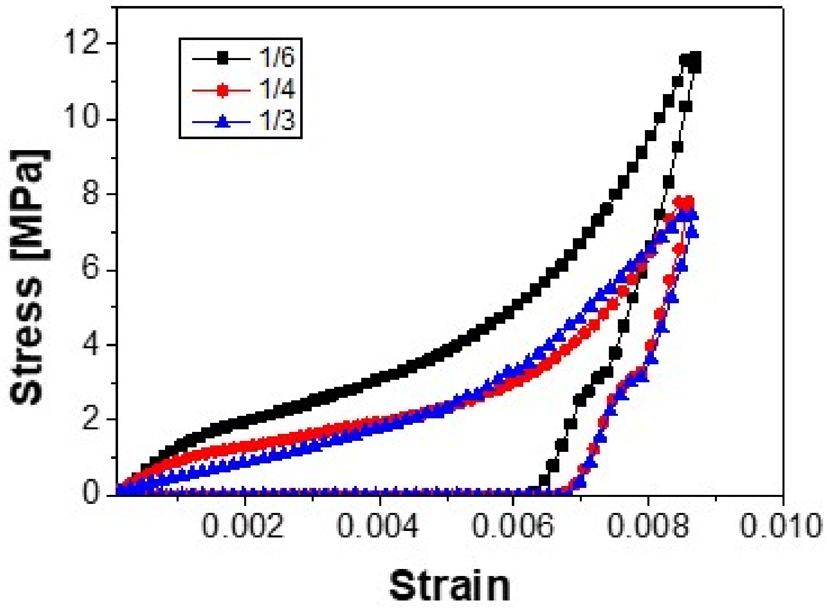

Fig. S5 Data of compression test of membranes. All membranes had a rectangular shape in size of 0.3 × 0.3 × 0.1 cm^3.

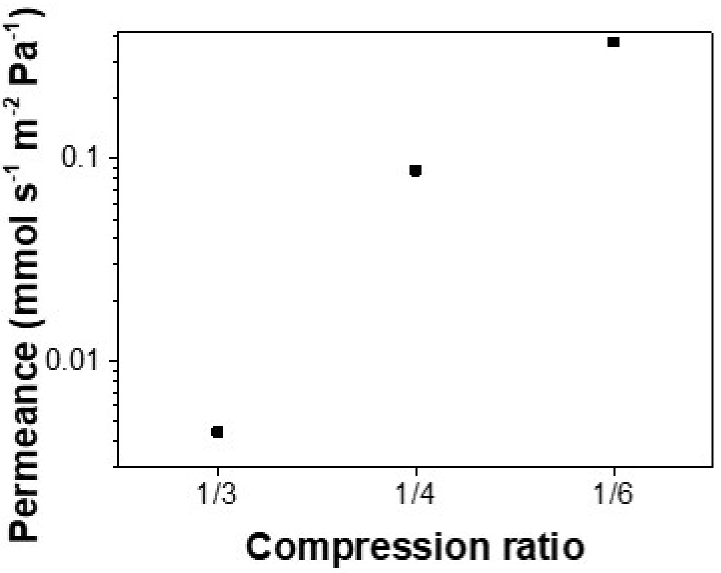

Fig. S6 Gas permeance of the VACNT membrane according to the compression ratio. Gas permeance was measured using a homemade bubble flow meter. After N2 was injected in the compressed CNT, the flow rate was measured at the moving speed of the bubble.

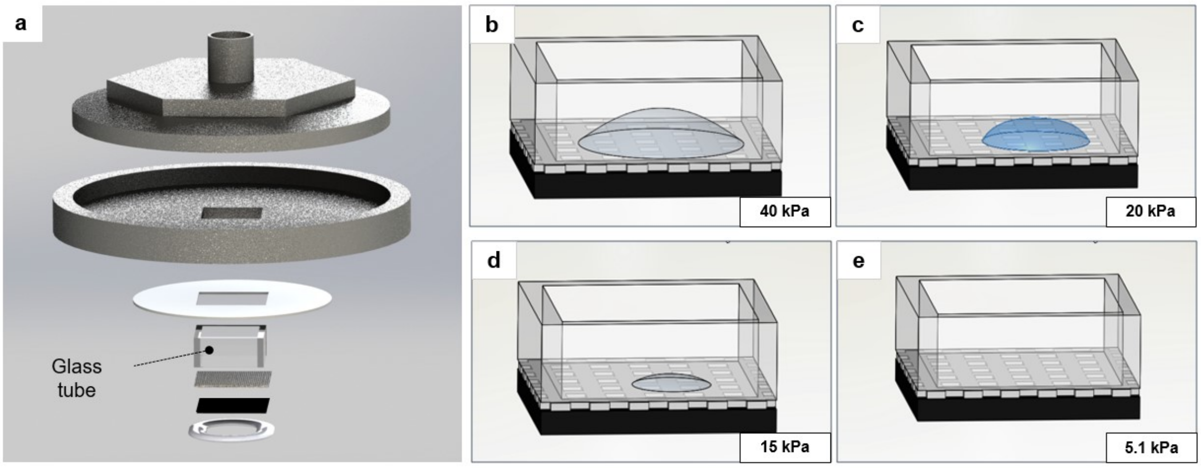

Fig. S7 Visualization of the water transudation and vaporization on the VACNT membrane assembly surface. (a) Schematic diagram of the setup and the viewing angle of the camera. (b-e) The image that replaced the actual shooting screen with a schematic, taken through the glass channel during the VMD operation. Each picture shows the situation under the pressure of 40 kPa, 20 kPa, 15 kPa, 5.1 kPa in order. The circular shape represents liquid water that has passed through the membrane.

An additional experiment was conducted to confirm how water passes through the membrane during the VMD experiment. In the setup (**fig. S7a**), a rectangular glass tube was installed in between the PE film and the aluminum mesh so that water passing through the membrane could be optically examined. Therefore, the porous plane, shown in the center of **Fig. S7b-e**, represents an aluminum filter serving as a substrate for the membrane.

As the vacuum is applied from atmospheric pressure, the feed water starts to pass through the membrane at some pressure (**fig. S7b**), and at the lower pressure, boiling occurs as the boiling point is lowered (**fig. S7c**). At far lower pressure, the water penetrated through the membrane disappears by vaporization (**fig. S7d**), and when it approaches 5.1 kPa, there is no liquid above the membrane (**fig. S7e**), suggesting that the water transuding from the membrane promptly vaporizes or the water starts to vaporize inside the membrane during the VMD experiment and passes the membrane as vapor.
